# Phase I randomized double-blind placebo-controlled single-dose safety studies of Bowman-Birk inhibitor concentrate

**DOI:** 10.3892/ol.2014.1855

**Published:** 2014-02-05

**Authors:** LILIE L. LIN, ROSEMARIE MICK, JEFFREY WARE, JAMES METZ, ROBERT LUSTIG, NEHA VAPIWALA, RAMESH RENGAN, ANN R. KENNEDY

**Affiliations:** 1 Department of Radiation Oncology, Perelman School of Medicine, University of Pennsylvania, Philadelphia, PA 19104, USA; 2 Department of Biostatistics and Epidemiology, Perelman School of Medicine, University of Pennsylvania, Philadelphia, PA 19104, USA

**Keywords:** Bowman-Birk inhibitor, soybean, cancer prevention, Bowman-Birk inhibitor concentrate

## Abstract

In previously performed animal studies and Phase I–II human trials, Bowman-Birk inhibitor concentrate (BBIC) appeared to be a promising cancer chemopreventive agent. The present study describes the results of two phase I randomized double-blind placebo-controlled trials performed in male subjects to assess the safety and toxicity of the original and new formulations of BBIC administered in a single dose as a suspension in orange juice. The dose of BBIC varied from 800–2,000 chymotrypsin inhibitor (CI) units. The BBI concentration in the serum samples collected from the subjects was analyzed by a dot-blot analysis procedure using the 5G2 monoclonal antibody, which is specific for reduced BBI. A total of 41 subjects were enrolled, 20 in the initial BBIC study and 21 in the second BBIC study. In these human trials, no clinically relevant changes in hematological or biochemical parameters were observed. Overall, BBIC was found to be well-tolerated. For these BBIC single-dose phase I trials, there was no dose-limiting toxicity for BBIC, even at the highest dose evaluated, and there were no apparent differences between the clinical trial results for the two formulations of BBIC. The bioavailability of BBI in the second clinical trial, which used the new BBIC formulation, was approximately 40 to 43% of the BBI bioavailability reached in the first clinical trial, which used the original BBIC formulation. The observed bioavailability difference was attributed to the different BBIC formulations used in these two clinical trials. These trials demonstrated that BBIC is safe when administered in a single dose of up to 2,000 CI units. Therefore, the results from the two trials indicate that a multi-dose trial of BBIC may be safely performed with doses of up to 2,000 CI units per day.

## Introduction

Increasing evidence indicates that individuals with a high dietary intake of soybean-derived products have low incidence and mortality rates from common cancers in the Western hemisphere, including cancers of the colon, breast and prostate (1). A number of different agents in soybeans may act as cancer chemopreventive agents in human populations (2). These agents include the soybean-derived protease inhibitor, Bowman-Birk inhibitor (BBI), inositol hexaphosphate (phytic acid), the sterol, β-sitosterol, and the isoflavone, genistein, which have been demonstrated to suppress the development of cancer in animal carcinogenesis assay systems. BBI has been shown to have the strongest anticarcinogenic activity in animal carcinogenesis model systems in comparison to other potential cancer chemopreventive agents in soybeans (2). BBI, as a purified compound and an extract of soybeans in which BBI has been concentrated, has been shown to suppress carcinogenesis in a wide variety of *in vivo* and *in vitro* carcinogenesis assay systems (3).

BBI is an 8-kDa soybean-derived protein containing 71 amino acids with two functional domains. One domain inhibits trypsin, the other inhibits chymotrypsin and several other serine proteases with chymotrypsin-like specificity, including elastase (4,5), cathepsin G (5,6) and chymase (7). BBI has been shown to have several therapeutic activities (reviewed in 8–10). BBI concentrate (BBIC) is a soybean extract enriched in BBI (11). It is believed that the chymotrypsin inhibitory activity of BBI conveys these therapeutic activities, therefore, the potency of BBIC is measured in chymotrypsin inhibitor (CI) units. One CI unit is defined as the amount of a substance required to inhibit 1 mg of bovine pancreatic chymotrypsin (11). Like BBI, BBIC inhibits trypsin and chymotrypsin and is anticarcinogenic, as measured by its ability to prevent malignant transformation *in vitro* and suppress carcinogenesis *in vivo* (reviewed in 3,9,11,12). In phase I clinical trials performed previously, no toxicity was observed when BBIC was orally administered in a single dose of up to 800 CI units in patients with premalignant lesions known as oral leukoplakia (13) or in daily doses of up to 800 CI units for 6 months in patients with benign prostatic hyperplasia (14). A subsequent phase IIa clinical trial in patients with oral leukoplakia demonstrated a dose-dependent reduction in oral lesion size after a one-month treatment with BBIC at doses of up to 1,066 CI units (15). In the clinical trial with benign prostatic hyperplasia patients, statistically significant decreases were observed in the serum prostate-specific antigen (PSA) level, serum triglyceride level and prostate volume following a 6-month treatment period with BBIC at doses of up to 800 CI units (14). BBIC tablets have also been administered to patients with active ulcerative colitis at a dose of 800 CI units per day for 12 weeks (16). In this study, the Sutherland Disease Activity Index (SDAI) was used to assess disease activity, response (index decrease >3) and remission (index <1 with no rectal bleeding). Favorable trends were observed in the rates of remission and clinical response, and no severe adverse events were observed. The results of the trial indicated a potential advantage over the placebo for achieving a clinical response and the induction of remission in patients with active ulcerative colitis, without apparent toxicity.

Based on the non-toxicity and positive clinical responses observed in the previous clinical trials, two additional clinical trials were performed for the present study, using single BBIC doses of up to 2,000 CI units to determine the pharmacokinetics and safety of BBIC administered orally as a suspension in orange juice (OJ). Males were chosen for these trials as it was predicted that this would be the beginning of a prostate cancer prevention program utilizing BBI as the prostate cancer chemopreventive agent. One of these trials used the original formulation of BBIC and the other trial used a new formulation of BBIC. The primary objectives were to determine i) the dose-limiting toxicities for single doses of BBIC and expansion of the range of doses tested in humans, ii) the recommended doses of BBIC for a subsequent phase I multiple*-*dose study, and iii) pharmacokinetic characterization of the original and new BBIC formulations.

## Materials and methods

Two sequential randomized, double-blind, placebo-controlled trials were performed in healthy male subjects (NCT00287833 and NCT00679094). The trials were approved by the Institutional Review Board at the University of Pennsylvania (Philadelphia, PA, USA) and all subjects provided informed consent. The subjects were male, aged 18–65 years and assessed to be in good health by physical exam, electrocardiography and standard hematological tests. The inclusion criteria consisted of an Eastern Cooperative Oncology Group performance status (17) of 0–2, the lack of chronic medical conditions, no evidence of psychiatric problems and a weight within 15% of the ideal body weight. The subjects were excluded if they had a history of heart disease, chemotherapy in the last 12 months, tobacco smoking, allergies or prior adverse reactions to soybeans or if they had a prior diagnosis of pancreatic or other gastrointestinal diseases. Those who reported taking more than two vitamin supplements or non-steroidal anti-inflammatory drug on a regular basis, and vegetarians and others with a large soy component to their diet were also excluded.

### BBIC

The original BBIC formulation was manufactured by Central Soya (Fort Wayne, IN, USA) according the procedure described previously (11). The new BBIC formulation was manufactured originally by Central Soya, but purified by Dynamic Extractions Ltd., (DE; Slough, Berkshire, UK) under direction from the National Cancer Institute (NCI) Division of Cancer Prevention (DCP) and supplied to the University of Pennsylvania School of Medicine Investigational Drug Service by the NCI Repository (McKesson Bioservices, Rockville, MD, USA). The contents of the original BBIC formulation have been described in detail previously (11). While the original BBIC formulation did contain low levels of normal food bacteria that are deemed non-pathogenic and are not expected to lead to adverse health effects, the NCI DCP produced a more purified formulation of BBIC that would not contain the normal food bacteria and represented a more concentrated version of BBIC. A more concentrated form of BBIC was expected to be considerably more useful in future human trials in which the total BBIC doses could be increased substantially. The company chosen to purify BBIC was DE, which is a UK specialist chromatography company. The DE product was called freeze-dried BBIC (BBIC-700). The final CI activity of the DE product was 562 CI units/g, while the original BBIC product was approximately 100 CI units/g (11). The microbiological content of the DE product was as follows: Aerobes <1 cfu/g (upper limit, 100 cfu/g); yeasts and molds <1 cfu/gram (upper limit, 100 cfu/g); *E. coli* and Salmonella were absent. The methods used in the preparation of freeze-dried BBIC were consistent with current good manufacturing principles. The new BBIC formulation from DE was stored in the refrigerator at 2 to 8°C. The original BBIC formulation was stored at room temperature.

### Study design and endpoints

Each study aimed to enroll a total of 20 healthy male volunteers, who were sequentially assigned to four different cohorts (drug levels), with five subjects per cohort. Subjects were recruited from the city of Philadelphia using print advertisements in local newspapers and posters around the campus of the University of Pennsylvania. One subject per cohort was assigned to receive a placebo instead of the active medication. Assignment to placebo or active medication was performed in a random and double-blind manner, the study pharmacist used a random number generator to assign treatments. The assigned treatment group was revealed at the completion of the trial. The following drug levels were investigated: 800, 1,200, 1,600 and 2,000 CI units. The first and second BBIC trials used the original formulation and the new formulation of BBIC, respectively. The subjects assigned to receive the placebo received 11.5 fl oz of OJ (Minute Maid Original 100% Orange Juice from Concentrate; Minute Maid, Sugar Land, TX, USA) with no additives. For subjects receiving BBIC, the measured dose of study medication was suspended in 11.5 fl oz of OJ from a single container. Both of the above ingredients were added to a subsequently sealed container and agitated until the suspension mixed uniformly. The total volume of the preparation was approximately 12 fl oz (350 ml). BBIC was administered orally to subjects in the form of a suspension at a concentration of 6% (w/v) in OJ. The CI activity is preserved in this formulation at this medication concentration for at least 3 h after suspension in the OJ, so the study medication was administered immediately after suspension in OJ.

### Subjects and sampling schedule

The subjects arrived at the Clincial and Translational Research Center (Philadelphia, USA) after fasting overnight. The subjects swallowed a single dose of BBIC suspension or placebo and immediately ate a defined low-fat breakfast. The subjects remained in the clinical research facility for the first 48 h of the study to facilitate the required frequent blood draws and to ensure that all subjects consumed the same low-fat diet. Subsequent to the first 48 h, the subjects returned to their homes, ate their normal diets without restrictions and then returned to the clinic for the required blood draws.

Blood and urine samples were obtained for BBI pharmacokinetic evaluation at the following times following completion of drug ingestion: 0 (immediately prior to BBIC administration), 0.5, 1, 2, 3, 4, 5, 6, 8, 10, 12, 24 and 48 h. Additional blood samples were obtained at 12, 24 and 48 h, and on or around days 7, 14, 21 and 28 for clinical blood chemistry and toxicity evaluation.

Serum for pharmacokinetic analysis was separated from blood cells and frozen at −80°C in 1-ml aliquots. Urine samples were obtained at the same times as blood samples. At each collection time, the subjects were directed to void their bladder. All urine collected during that time interval was pooled, total volume recorded and a sample was frozen at −20°C for pharmacokinetic analysis.

Amylase, lipase and low density lipoprotein/high density lipoprotein (LDL/HDL) were also assessed at times 0h, 12 h, 1 week and 4 weeks. Safety and toxicity were scored using the NCI Common Toxicity Criteria scoring system version 2.0. Statistical analysis consisted of tabulation of graded toxicities by dose level.

### Reagents used for BBI measurement

A mouse monoclonal antibody, designated 5G2, was used as a primary antibody for BBI measurement by a dot-blot analysis in this study. The 5G2 antibody was produced and characterized as previously described (18). A horseradish peroxidase-conjugated goat anti-mouse IgG2b antibody was purchased from Southern Biotechnology Associates (Birmingham, AL, USA) and used as a secondary antibody for the BBI measurement. BBI was purchased from Sigma Chemicals (St. Louis, MO, USA) and radio-chemically reduced by exposure to ^137^Cs γ-rays under anoxic conditions in a buffer containing 100 mM formate and 10 mM phosphate buffer (PB; pH 5.5), as previously described (18). The reduced BBI was diluted to a concentration of 50 μg/ml and used as a stock solution of BBI standard antigen for BBI quantitation. Enhanced chemiluminescence (ECL) reagent was purchased from GE Healthcare (Piscataway, NJ, USA) for dot visualization in the dot-blot analysis.

### BBI measurement by dot-blot analysis

The BBI in serum samples was measured using a dot-blot analysis procedure. The serum samples were diluted in a buffer containing 70% 10 mM sodium PB (pH 7.5) and 30% absolute ethyl alcohol. For each dot-blot analysis, the BBI standard was serially diluted in a buffer containing 70% 10 mM PB (pH 7.5) and 30% absolute ethyl alcohol to BBI concentrations of 0 (baseline), 10, 30, 50, 100, 150 and 200 ng/ml and analyzed along with the serum samples to generate a standard curve for BBI quantitation.

To perform the dot-blot analysis, three 10-μl aliquots of each sample or BBI standard were spotted onto an immobilion-PSQ membrane (Millipore, Billerica, MA, USA), with each membrane containing the entire set of serum samples from two or three subjects in addition to the BBI standards. The membranes were allowed to dry completely at room temperature. The membranes were rinsed with 10 mM PB (pH 7.5) for 2 min, blocked with 5% milk for 30 min, rinsed with PB three times for 5 min each and incubated with the primary antibody for 1 h. Following incubation, the membranes were rinsed again with PB three times for 5 min each and incubated with the secondary antibody for 1 h. The membranes were washed with PB and incubated with ECL reagent for 1 min, and then exposed to X-ray film.

The integrated density of each spot on the membrane was obtained by scanning the X-ray films using ImageJ software (Sigmaplot version 12.0) from the National Institutes of Health (http://rsbweb.nih.gov/ij/index.html) following background subtraction. For each dot-blot analysis, triplicate values of each BBI standard were averaged and a standard curve was established by a linear regression analysis using the BBI concentration as the independent variable and the averaged integrated density as the dependent variable. The BBI concentration in each serum sample was determined using the standard curve generated on the same dot-blot membrane.

### Pharmacokinetic and statistical analysis

The serum BBI levels were analyzed using pharmacokinetic function macros developed for Microsoft Excel (http://www.boomer.org/pkin/soft.html). The data for the area under the curve (AUC) in each clinical trial were analyzed by linear regression analyses and compared among different treatment groups by one-way ANOVA followed by Tukey’s test. The AUC data from the two clinical trials were further analyzed by two-way ANOVA using the BBIC dose and BBIC formulation (original vs. new) as the independent variables. P<0.05 was considered to indicate a statistically significant result.

## Results

### First BBIC study

A total of 37 patients were screened and a total of 20 subjects were enrolled in the initial BBIC study between December 2005 and March 2007. The characteristics of the patients enrolled are listed in Table I. No subject was lost to follow-up. Adverse events were observed in placebo- and BBIC-treated subject groups at approximately equal frequency. A total of 50 adverse events were observed in the 16 BBIC-treated subjects (mean, 3.125 per subject) vs. 13 in the 4 subjects receiving the placebo (3.25 per subject). The rates of adverse events did not increase with dose, and in the majority of cases were lower in the highest dose group than in the other groups. The exceptions to this were incidents of grade 1 hyperglycemia [6 reported incidents in the 16 treated subjects (3 in subjects receiving 2,000 CI units) vs. 1 incident in a subject receiving placebo] and a single report of hyperkalemia in a subject receiving 2,000 CI units (Tables II and III). The only grade 3 adverse event was a high alanine aminotransferase (ALT) level in a subject who received 800 CI units. The subject’s ALT levels were observed to be normal during the inpatient portion of the study (48 h post-ingestion time period). During the subject’s one week follow-up visit, the grade 3 high ALT was observed, but by the second week, the ALT had resolved to a normal level.

### Second BBIC study

A total of 34 subjects were screened and a total of 21 subjects were enrolled in the second BBIC trial between June 2007 and March 2009. No subject was lost to follow-up. A dosing error occurred for one subject, who received 1,500 CI units BBIC instead of 1,200 CI units BBIC. This subject’s data were reported separately in this analysis and the subject was replaced by an additional patient in the 1,200 CI units BBIC dose cohort. Hence, there were a total of 21 subjects in the second study instead of the initially intended 20 subjects. No other dosing anomalies occurred.

No serious adverse events were reported during the study. The only grade 2 toxicities reported were abnormal triglyceride and aspartate aminotransferase (AST) levels and hypoglycemia. The single occurrence of a grade 2 abnormal triglyceride level occurred in a patient receiving the placebo (Table IV). The single occurrence of a grade 2 abnormal AST level occurred in a patient in the 1,600 CI units BBIC dose level (Table V). Ten occurrences of grade 2 hypoglycemia were experienced at 800 CI units BBIC (3 subjects), 1,200 CI units BBIC (3 subjects), 1,600 CI units BBIC (2 subjects) and 2,000 CI units BBIC (2 subjects), and therefore did not appear to be correlated with dose.

### Pharmacokinetic characterization of the original and new BBIC formulations

**Figure 1:**
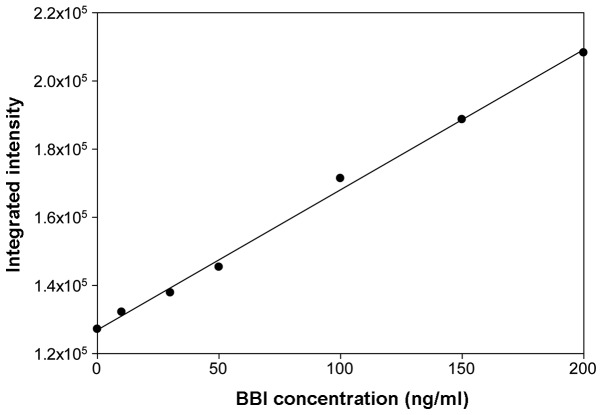
A representative standard curve of BBI. The BBI standard was serially diluted in a buffer containing 70% 10 mM phosphate buffer (pH 7.5) and 30% absolute ethyl alcohol to reach final BBI concentrations of 0 (baseline), 10, 30, 50, 100, 150 and 200 ng/ml. For each dot-blot assay, each BBI standard solution was spotted onto an immobilion-PSQ membrane in triplicate and analyzed in addition to the serum samples to generate a standard curve for BBI quantitation. BBI, Bowman-Birk inhibitor.

**Figure 2:**
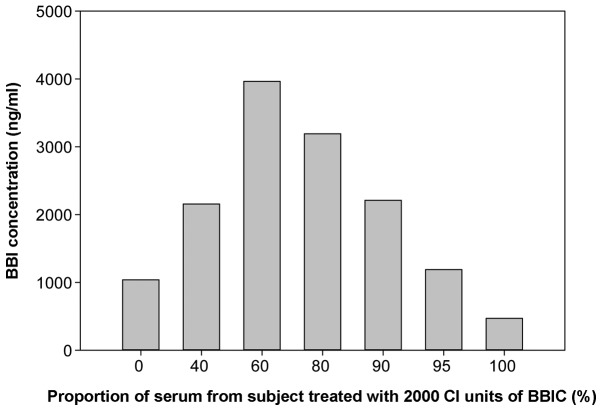
‘Hook effect’ observed in dot-blot analysis. A serum sample from a subject treated with 2,000 CI units of BBIC was mixed with a serum sample from a placebo treated subject in proportions of 0, 40, 60, 80, 90, 95 or 100%. Each mixture was diluted 1:200 with a buffer containing 70% 10 mM phosphate buffer (pH 7.5) and 30% absolute ethyl alcohol, spotted onto an immobilion-PSQ membrane and analyzed in addition to serially diluted BBI standards. BBI, Bowman-Birk inhibitor; CI units, chymotrypsin inhibitor units.

The BBI concentration in the serum samples collected from subjects orally administered with BBIC at doses of up to 2,000 CI units was analyzed by a dot-blot analysis procedure using the 5G2 monoclonal antibody, which is specific for reduced BBI. Based on the signal responses of the BBI standards included in the assay, the dot-blot analysis was linear in a BBI concentration range of 0–200 ng/ml (Fig. 1). Preliminary analyses of 1:200 diluted serum samples produced lower BBI results in the dot-blot analyses for the subjects who received the highest dose of BBIC than for the subjects in the other treatment groups (data not shown), which indicated the presence of a hook effect of falsely low values in immunoassays when an excess of antigen affects the binding capacity of the detection antibody (18–20). To confirm the presence of the hook effect, a serum sample from a subject treated with the highest dose of BBIC (2,000 CI units) was mixed with a serum sample from a subject treated with placebo in various proportions and subjected to the dot-blot analysis. The results indicated that the measured BBI values increased with the proportion of serum from the BBIC treated subject up to 60% and then declined (Fig. 2). The results confirmed the presence of a hook effect in the dot-blot analysis for the 1:200 diluted serum samples from the subject in the highest BBIC dose group.

To avoid a possible hook effect on the serum BBI measurement, the serum samples from subjects in the two highest BBIC dose groups were diluted 1:500 for the dot-blot analysis. The serum samples from subjects treated with placebo or BBIC at the two low doses (800 and 1,200 CI units) were diluted at 1:200 for the dot-blot analysis, since the BBI concentration in these samples was not predicted to be high enough to cause a hook effect.

**Figure 3:**
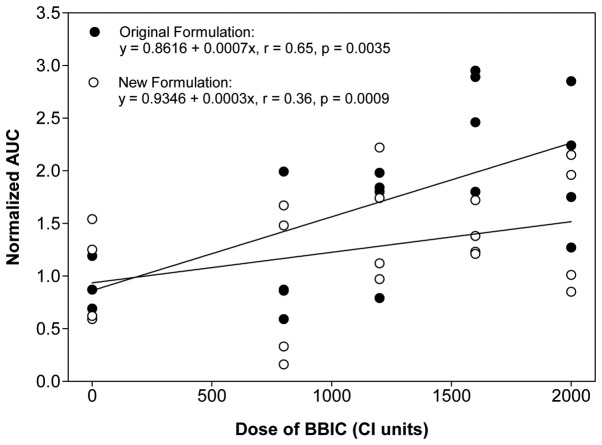
Regression analysis of serum BBI results. The correlation between the serum BBI results expressed as normalized AUC values and BBIC dose was evaluated by a linear regression analysis for each clinical trial using the BBIC dose as the independent variable and AUC value as the dependent variable. BBI, Bowman-Birk inhibitor; CI units, chymotrypsin inhibitor units; AUC, area under the curve; BBIC, BBI concentrate.

**Figure 4:**
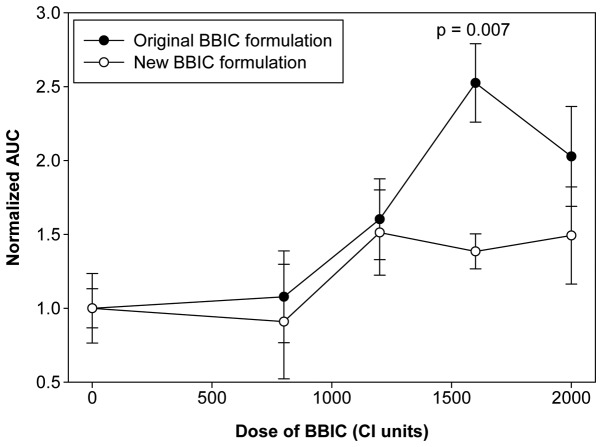
Dose-response curve of serum BBI results. The mean normalized AUC values for serum BBI levels of different treatment groups were plotted against the BBIC dose and compared by two-way ANOVA followed by Tukey’s test. The statistical significance for the comparison of serum BBI results between the two clinical trials at each BBIC dose is indicated by the P-value shown in the graph. BBI, Bowman-Birk inhibitor; CI units, chymotrypsin inhibitor units; AUC, area under the curve; BBIC, Bowman-Birk inhibitor concentrate.

For the first phase I trial, the normalized AUC for the serum BBI level was moderately correlated with the BBIC dose, with a correlation coefficient of 0.65 and a slope value of 0.0007 (Fig. 3). The mean normalized AUC values for subjects treated with placebo or BBIC at doses of 800, 1,200, 1,600 or 2,000 CI units were 1.00±0.13, 1.08±0.31, 1.60±0.27, 2.53±0.27 and 2.03±0.34, respectively (Fig. 4). The difference among the treatment groups was statistically significant (P=0.006 by one-way ANOVA).

For the second phase I trial, the normalized AUC for the serum BBI level was only weakly correlated with the BBIC dose, with a correlation coefficient of 0.36 and a slope value of 0.0003 (Fig. 3). The mean normalized AUC values for the subjects treated with placebo or BBIC at doses of 800, 1,200, 1,600 or 2,000 CI units were 1.00±0.24, 0.91±0.39, 1.51±0.29, 1.39±0.12 and 1.49±0.33, respectively (Fig. 4), and the difference was not statistically significant (P=0.446 by one-way ANOVA).

To determine whether the dose-responses of the serum BBI levels were different between the first clinical trial, which used the original BBIC formulation, and the second clinical trial, which used the new BBIC formulation, the AUC data obtained in the two clinical trials were analyzed by a two-way ANOVA. The results indicate that the serum BBI level expressed as the AUC was significantly affected by the BBIC dose (P=0.022) and the BBIC formulation (P=0.031) received by the subjects. The mean normalized AUC value for BBIC-treated subjects (all doses combined) was 1.808 and 1.325 for the first and second clinical trials, respectively, and the difference was statistically significant (P=0.031). After subtracting the baseline normalized AUC value of 1 for the placebo treatment group, the net increases in the mean normalized AUC value for BBIC treated subjects in the first and second clinical trials were 0.808 and 0.325, respectively.

## Discussion

Two phase I randomized double-blind pharmacokinetic and safety trials were conducted using two different formulations of BBIC, a candidate chemopreventive agent, administered orally as a suspension in OJ. These clinical trials were initiated in preparation for a prostate chemoprevention program in prostate cancer at the University of Pennsylvania. The main objectives of the pharmacokinetic analyses were to measure and characterize the levels of BBI in serum and urine following a single dose of BBIC or placebo in healthy male subjects; these analyses will be reported separately. No serious adverse events were observed. No clinically significant laboratory abnormalities were reported for any dose level.

Four human trials utilizing BBIC have been completed previously (13–15,22–24). Phase I and phase IIa studies of BBIC in patients with oral leukoplakia were also performed (13,15,22,23). The results from these trials indicated no toxicity from BBIC at any dose level studied. Over the dose range of 200–1,000 CI units per day, BBIC caused a reduction of total oral leukoplakia lesion size that was linearly correlated with increase in dose. The compound was well-tolerated with no evidence of laboratory, symptomatic or clinical side-effects (23). A randomized, double-blind trial of BBIC in patients with benign prostatic hyperplasia (BPH) was also performed (14,24). This trial involved 6 months of BBIC treatment involving dosage levels of 100–800 CI units per day in oral tablet form. There was no dose-limiting toxicity of BBIC observed in this study. For the BBIC single-dose phase I trials reported in the present study, the dose range chosen was higher than any that had been used previously in BBIC human trials (800–2,000 CI units). No dose-limiting toxicity was observed for BBIC, even at the highest dose evaluated (2,000 CI units), and the results from the two trials were comparable for the studies involving two different formulations of BBIC. Thus, the results from the two trials indicate that a multidose trial of BBIC may be performed with doses of up to 2,000 CI units per day.

The pharmacokinetic studies using urine samples from previously completed clinical trials have shown that BBI is excreted rapidly in the urine between 3 and 12 h after BBIC administration (13). Linear regression analyses of the BBI results demonstrated dose-dependent increases in mean BBI concentration, peak BBI concentration, peak minus mean BBI concentration and the peak to mean ratio of BBI concentration in the urine samples of subjects following BBIC treatment (14). These results indicate that BBI is absorbed systemically in human subjects following oral administration of BBIC. In the present study, serum BBI levels were determined for subjects enrolled in two new clinical trials using the original and new BBIC formulations. The results demonstrate that the AUC value for the serum BBI level was significantly correlated with the dose of BBIC received by the subjects in the two trials, however, the slope of the linear regression line for the first trial (slope=0.0007) was more than twice as steep as the slope of the linear regression line for the second clinical trial (slope=0.0003). In addition, the mean normalized AUC value for the serum BBI level in the BBIC-treated subjects was significantly higher in the first clinical trial than in the second clinical trial. Based on the ratio of regression line slopes between the two clinical trials (0.0003/0.0007=0.4286) and the ratio of the net increase in the mean normalized AUC values (0.325/0.808=0.4022), the bioavailability of BBI in the second clinical trial was approximately 40 to 43% of the BBI bioavailability reached in the first clinical trial. Since the two clinical trials were performed using the same experimental design, with the exception of the BBIC formulation, the differences observed are attributable to the different BBIC formulations used in these two clinical trials.

While the CI activity in the original and new formulations of BBIC appeared to be comparable, there are numerous variables that may affect the biological activities of BBI/BBIC, as previously discussed (11). Two of these factors are the refrigeration and freezing of BBIC samples, which result in a reduced ability of the samples to affect radiation-induced transformation *in vitro* (11). The new formulation of BBIC was exposed and maintained under refrigerated and frozen conditions during the purification procedure used by DE or the subsequent storage of the new product, while it is recommended that the original formulation of BBIC should not be exposed to either refrigerated or frozen conditions. It is likely that the temperatures, or the new solvents (ethyl acetate and methanol) used by DE in the production of the new formulation of BBIC, may have altered the bioavailability of BBI compared to the BBI in the original formulation of BBIC.

Prior to the BBIC clinical trials in humans, information about the absorption, distribution and excretion of BBI was primarily based on animal studies utilizing radiolabeled BBI. These studies indicated that approximately half of the BBI administered orally is excreted in the feces in an unaltered form, whereas the remainder enters intestinal epithelial cells (25) or crosses the intestinal lumen via a paracellular mechanism (26). In animal studies, BBI is able to survive the digestive process, reach the colon in an active form and is capable of interacting with proteases in the same manner as expected for BBI (26,27). The measurement of BBI in biological samples by immunoassay has proven to be technically challenging. BBI is readily detectable using monoclonal antibodies that have been generated by Brandon *et al* (28) in food samples (29) or in human serum and urine samples spiked with purified BBI in its native form (30). Oral administration of BBI results in a form of BBI in the bloodstream and urine that cannot be detected with the antibodies against BBI in its native form (18), despite the fact that the BBI appearing in blood and urine following oral intake has the same molecular weight and the same ability to inhibit trypsin and chymotrypsin as BBI (26). Since it is necessary to use antibodies reactive with reduced BBI to detect BBI in blood and urine samples from subjects following BBI oral intake (18,31), it is assumed that BBI is present in a reduced form in body fluids. Pharmacokinetic studies of BBI have previously been performed in rodents, dogs and humans with antibodies that react with reduced BBI (reviewed in 32). As part of subchronic toxicity studies of BBIC in rats and dogs, serum concentrations of BBI were measured using one of the antibodies that reacts specifically with reduced BBI, known as 5G2 (18), by a dot-blot method. As summarized previously (32), the serum BBI level was 32–48% higher in rats treated with oral daily doses of 500 or 1,000 mg/kg BBIC for 3 months and 35–50% higher in dogs treated with oral daily doses of 500 or 1,000 mg/kg BBIC for 45 or 89 days compared to their respective control groups. The highest dose of BBIC administered to human subjects in the present study was at least one order of magnitude below the dose used in the previous rat and dog toxicity studies on a kg-body weight basis. However, the magnitude of increase in the mean normalized AUC value for the serum BBI level of the highest BBIC dose groups in the present study was of the same order of magnitude as previously observed in animal toxicity studies, indicating that there may be an upper limit for BBI absorption following oral administration. Based on the shape of the dose-response curves of the serum BBI level (Fig. 4), the serum BBI level appeared to reach a plateau at the dose of 1,600 CI units, and further increases in the BBIC dose did not result in an additional increase in the BBI level in the circulation. This may have practical implications for future clinical trials using BBIC or other soybean-based dietary supplements with BBI as the main active ingredient.

The results from the first and second trials of BBIC utilizing the original and new BBIC formulations indicate that a dose-limiting toxicity for BBIC was not observed up to a dose of 2,000 CI units. Therefore, it is proposed that a multidose BBIC study may be extended up to a high dose of 2,000 CI units per day.

## Figures and Tables

**Table I:** Characteristics of patients in each BBIC study.

	First BBIC study		Second BBIC study	
		
Characteristic	BBIC	Placebo	BBIC	Placebo
Caucasian, n	13	4	9	3
African descent, n	1	0	5	0
Asian or Pacific Islander, n	1	0	2	1
Hispanic, n	1	0	0	0
Mixed ethnicity, n	0	0	1	0
Mean age, years	27.9	33.3	30.2	34.9

BBIC, Bowman-Birk inhibitor concentrate.

**Table II:** Maximum toxicity in BBIC-treated subjects (combined) in the first BBIC trial (n=16).

	Maximum toxicity grade				
	
Toxicity type	0	1	2	3	4
ALT, n	14	1	0	1	0
AST, n	12	3	1	0	0
Bilirubin, serum-high (hyperbilirubinemia), n	15	1	0	0	0
Calcium, serum-low (hypocalcemia), n	14	2	0	0	0
Cholesterol, serum-high (hypercholesteremia), n	14	2	0	0	0
Glucose, serum-low (hypoglycemia), n	2	10	4	0	0
Glucose, serum-high (hyperglycemia), n	10	6	0	0	0
Headache, n	14	2	0	0	0
Hemoglobin, n	15	1	0	0	0
Infection, cold-sore (lip), n	15	0	1	0	0
Leukocytosis, n	12	4	0	0	0
Potassium, serum-high (hyperkalemia), n	15	1	0	0	0
Sodium, serum-high (hypernatremia), n	11	5	0	0	0
Triglyceride, serum-high (hypertriglyceridemia), n	14	2	0	0	0

BBIC, Bowman-Birk inhibitor concentrate; ALT, alanine aminotransferase; AST, aspartate aminotransferase.

**Table III:** Maximum toxicity in placebo-treated subjects in the first BBIC trial (n=4).

	Maximum toxicity grade				
	
Toxicity type	0	1	2	3	4
Albumin, n	3	1	0	0	0
ALT, n	4	0	0	0	0
AST, n	3	1	0	0	0
Bilirubin, serum-high (hyperbilirubinemia), n	2	2	0	0	0
Calcium, serum-low (hypocalcemia), n	2	2	0	0	0
Cholesterol, serum-high (hypercholesteremia), n	3	1	0	0	0
Glucose, serum-low (hypoglycemia), n	1	2	1	0	0
Glucose, serum-high (hyperglycemia), n	3	1	0	0	0
Headache, n	4	0	0	0	0
Hemoglobin, n	3	1	0	0	0
Infection, cold-sore (lip), n	4	0	0	0	0
Leukocytosis, n	4	0	0	0	0
Potassium, serum-high (hyperkalemia), n	4	0	0	0	0
Sodium, serum-high (hypernatremia), n	4	0	0	0	0
Triglyceride, serum-high (hypertriglyceridemia), n	3	1	0	0	0

BBIC, Bowman-Birk inhibitor concentrate; ALT, alanine aminotransferase; AST, aspartate aminotransferase.

**Table IV:** Maximum toxicity in placebo-treated control subjects for the second BBIC trial (n=4).

	Maximum toxicity grade				
	
Toxicity type	0	1	2	3	4
Alkaline phosphatase, n	3	1	0	0	0
AST, n	3	1	0	0	0
Calcium, serum-low (hypocalcemia), n	4	0	0	0	0
Calcium, serum-high (hypercalcemia), n	4	0	0	0	0
Creatinine, n	4	0	0	0	0
Dizziness, n	4	0	0	0	0
Cholesterol, serum-high (hypercholesteremia), n	2	2	0	0	0
Glucose, serum-low (hypoglycemia), n	0	4	0	0	0
Glucose, serum-high (hyperglycemia), n	1	3	0	0	0
Hemoglobin, n	3	1	0	0	0
Hemorrhage, GU - Bladder, n	3	1	0	0	0
Hemorrhage, GU - Urinary NOS, n	4	0	0	0	0
Lipase, n	4	0	0	0	0
Neutrophils/granulocytes (ANC/AGC), n	4	0	0	0	0
Potassium, serum-low (hypokalemia), n	4	0	0	0	0
Potassium, serum-high (hyperkalemia), n	4	0	0	0	0
Sodium, serum-high (hypernatremia), n	4	0	0	0	0
Triglyceride, serum-high (hypertriglyceridemia), n	1	2	1	0	0

BBIC, Bowman-Birk inhibitor concentrate; AST, aspartate aminotransferase; NOS, nitric oxide synthase; ANC, absolute neutrophil count; AGC, absolute granulocyte count; GU, genitourinary.
